# Supplementary material for: Patterns of Intron Gain and Loss in Fungi
Source: PLoS Biol. 2004 Nov 30;2(12):e422. doi: 10.1371/journal.pbio.0020422 (PMC532390; doi:10.1371/journal.pbio.0020422)
Supplement: Table S1 — Also available at http://genes.mit.edu/NielsenEtAl/. (4.3 MB ZIP). [file pbio.0020422.st001.zip › NielsenEtAl/html/177.html]

AN3749.1.NCU08115.1.MG09306.1.FG07112.1


```
 CLUSTAL W (1.82) Multiple Sequence Alignments - Introns Inserted


Sequence 1: MG09306.1	1151 aa
Sequence 2: FG07112.1	1105 aa
Sequence 3: NCU08115.1	1145 aa
Sequence 4: AN3749.1	1091 aa
Alignment Length: 1200 aa
Number Identitical Residues: 493 aa
Alignment Score (without introns) 23869


MG09306.1 	MAGPSGAAPKKQASLLGFFTPKTVNGLKRPTSSRPGTSEEDEERTADSSPSSRDNVRKRP
NCU08115.1	MAGPSRLPDKKQASISSFFTPRNTSPLVNLSQNASKKPPPAESKSSKSTS-----SRKRP
FG07112.1 	MAPTPKPGEKKQQSLTSFFTPKTVNGLAAIFEKQ--SRAQADKSQSPSKDITGLPSRKRA
AN3749.1  	MPLPS----SQPSASSSPNLKRKQPTISSFFTKKPQAPKQSTSNEGPAPIDNDSEITDKL
          	*. ..    .:  :  .    :.   :     . .       .  . :   .     .: 

MG09306.1 	LEQDPDAGNTRRPRASKRAKNVVIDDEDDEHDNDDDDDDFKLDAEAEADPEPVVAADDTA
NCU08115.1	EPQTDDS-EDDVPRDAKRRRS---NGPSAATDTEDAVASLKLSS----------------
FG07112.1 	VEDTEKENEEPKP-TSKRAR------ADDAVKDPGTSTFFSKSKDGEQE---------TI
AN3749.1  	AEDDEEDIVAPVPKRTKSNGS----LTVNRPQSPKAKSVSRVEQES--------------
          	  :  .      *  :*   .          .          . ..              

MG09306.1 	GVSQSSSISASRAGRYIYDESQPKGQAKTDENGDALDAATLRKKRE-LHDKFVKKLGHPD
NCU08115.1	---------SSRTERYALNSSRPS----QDEQEKEEDVAERKKKEE-LHRKFVKKLGHPD
FG07112.1 	GTRTER---YRYDGSSVWNTQDVDTNAGSSQNNDE-DAEERRQHQE-LHKKFVKKLGHPD
AN3749.1  	---------SQRTELSKFASSPAIETEGNEATELDGSAKVRQQEREKLHQRFVRKLGGPD
          	                    .        .      ..   ::..*.** :**:*** **

MG09306.1 	AMSRWGNAGRGEQETGAQEEEEDGDAAEEEPPPPPKGKKKGAKSG-KLTPMELQFLEIKR
NCU08115.1	SMFSYR--QRDTESAAVEGEGEEGEDDEEEPAPKTTAKKKGAKTG-KLTPMELQFLEIKR
FG07112.1 	ALVSRRNVQDAAPVG--EDEDADADGAEEEAPAPAKTKKKGSKTG-KLTPMEIQFLEIKR
AN3749.1  	CLVGIG--RNCVGETTSIEEAAEGDEDDETPQPVQPKGKAGKKGGGKLTPMEKQVIEIKK
          	.:                 *  :.:  :* . .     * * * *.****** *.:***:

MG09306.1 	KHMDTVLIVEVGYKFRFFGEDARIAGKELSIVCIPGKFRYDEH~PSEAHLDRFASASIPV
NCU08115.1	KHMDTLLIVEVGYKFRFFGEDARIAARELSIVCIPGKFRYDEH~PSEAHLDRFASASIPV
FG07112.1 	KHMDTVLIVEVGYKFRFFGDDARIAAKELSIVCIPGKMRYDEH1PSEAHIDRFASASIPV
AN3749.1  	KHMDTILLIEVGYKFRFFGEDARIAAKELSIVCIPGKFRYDEH1PSEAHLDRFASASIPV
          	*****:*::**********:*****.:**********:***** *****:**********

MG09306.1 	HRLPVHAKRLVAAGHKVGVVRQIETAALKKAGDNRNAPFVRKLCEVYTKGTYIDEMGEMD
NCU08115.1	HRLPVHAKRLVAAGYKVGVVRQIETAALKKAGDNRNAPFVRKLTNVYTKGTYIDETGELD
FG07112.1 	HRLPVHAKRLVAAGHKVGVVRQIETAALKKAGDNRNTPFVRKLTNLYTKGTYIDENGELD
AN3749.1  	QRLHVHVKRLVAAGHKVGVVRQLETAALKAAGDNRNAPFVRKLTNVYTKSTYIDDIESLE
          	:** **.*******:*******:****** ******:****** ::***.****:  .::

MG09306.1 	A-QTGASGAHSGGYLLCLTETAAKGSGTDEKVDVGLVAVQPATGDIIYDSFEDGFMRSEI
NCU08115.1	Q-PGETTGASSGGYLLCLTETPAKGMGTDEKVNVGIIAVQPATGDIIYDEFEDGFMRREI
FG07112.1 	--QSGGSGAPSGGYLLCITESKAKGSGTDEKVNVGIIAVQPATGDIIYDHFEDGFMRSEI
AN3749.1  	GSTAGASGASATGYILCITETNARGWGNDEKVHVGIVAVQPTTGDIVYDEFDDGFMRSEI
          	 :    :** : **:**:**: *:* *.****.**::****:****:** *:***** **

MG09306.1 	ETRLLHISPCELLIVGQLSKATEKLVKHLSGSASNVFGDRTRVERVAKGKTTPAEASSHV
NCU08115.1	ETRLLHISPCEFLIVGDLSKATDKLIQHLSGSSTNVFGDKSRVERVPKSKTMAAESYSNV
FG07112.1 	ETRLLHISPCEFLIVGDLTKGTDKLVQHLSGSSTNVFGDRSRVERVPKEKTMAADAYSHV
AN3749.1  	ETRLLHIAPCEMLIVGELSKATEKLVQHLSGSKMNVFGDKVRVERAPKAKTAAAESHSHV
          	*******:***:****:*:*.*:**::*****  *****: ****..* ** .*:: *:*

MG09306.1 	TKFYAGKLKGS--TQDDRAAALLDKVLNLPEPVTLCLSAMITHLTEFGLEHIFDLTKYFQ
NCU08115.1	TDFYAGKAKD----SDERSAALLNKVLKLPEAVMICLSAMITHLTEYGLQHIFDLTKYFQ
FG07112.1 	TQFYADKLKDN--AQDETAAALLGKVLKLPEPVTICLSAMINHLQEYGLEHIFDLTKYFQ
AN3749.1  	SSFYAEKMKSADAADDEVASNLLQKVLGLPDQVTICLSAMIKHMTEYGLEHVLQLTKYFQ
          	:.*** * *. .::.*: :: ** *** **: * :******.*: *:**:*:::******

MG09306.1 	SFSTRSHMCINGTTLESLEVYRNSTDHTEKGSLLWALDKTRTRFGQRMLRKWLGRPLLDK
NCU08115.1	SFSTRQHMLINGTTLESLEVYRNATDHSEKGSLLWALDKTHTRFGQRLLRKWIGRPLLDQ
FG07112.1 	SFSTRSHMLINGTTLESLEVYRNSTDHSEKGSLFWALDKTLTRPGQRLLRKWVGRPLLDQ
AN3749.1  	HFSSRSHMLLNGNTLTSLEIYQNQTDYSSKGSLFWTLDRTQTRFGQRMLRKWVGRPLLDR
          	 **:*.** :**.** ***:*:* **::.****:*:**:* ** ***:****:******:

MG09306.1 	ERLDDRVAAVEELFENRNGPQVEKLQKLLSSIKTDLERSLIRIFYGRCTRPELLAVLQTL
NCU08115.1	QRLEERVSAVEELLNNQSTAKVDKLVNMLKSIKADLERSLIRIYYGKCTRPELLSTLQTL
FG07112.1 	ELLEARLNAVEELLNKQSTAPVSQLESLLANTKTDLERSLIRIYYGKCTRPELLSVLQAL
AN3749.1  	RQLEDRVNAVEELKDFRNVVMVERIKGLLGKIKHDLEKGLIRIYYG--------------
          	. *: *: ***** : :.   *.::  :* . * ***:.****:**              

MG09306.1 	Q~RIAVEYIVVKEPSQTGFKSNLVSEALASLPRIREIVVSYLNRINPDAARKNDKYEFFR
NCU08115.1	Q~KISFEYARVKSPADTGFSSTLLTSAIMTLPSISPMVTAHLSKINAEAARKDDKYAFFL
FG07112.1 	Q~RVASYYSTIKAPSDAPFSSPLLSEAICSLPQILDTVVSYLERINLVAARKDDKYGFFR
AN3749.1  	K0MIAQEFADIESPADTGFSSPAISQAIMSLPTILKDVVFFLNKINMHAARNDDKYEFFR
          	:  ::  :  :: *::: *.*  ::.*: :** *   *. .*.:**  ***::*** ** 

MG09306.1 	DESDDTGDDGEDEITTQKMSIAAVEQELDAHRSDAAATLGRKKAVDYVTVAGIEYLIEVP
NCU08115.1	EQHET------EDISEHKLGIAAVEQDLDEHRSEAAKDLGKKVPVNYVTVAGIEYLIEVP
FG07112.1 	DEFQT------EDMQDHQLGIAHVEHELDGHRAVAAEKI-KKKMVDYVTVAGIEFLIEVP
AN3749.1  	EEEET------EEISEHKLGIGAVEHELEEHRPVAGEAL-GKKMVTYVSVAGIDYLVEVE
          	:: :       :::  :::.*. **::*: **. *.  :  *  * **:****::*:** 

MG09306.1 	N--TEIRKVPASWAKISGTKKLSRFHTPEVVRLIAERDQHKEALAAACDAAFKAMLASIA
NCU08115.1	N--TDLKRVPASWAKISGTKKVSRFHTPTVLRLIAERDQHKESLASACDQAFSDLLSQIA
FG07112.1 	N--ADIKHVPASWAKISGTKKLSRFHTPEVLRFITERDQHREALAAACDKAFKDLLASIA
AN3749.1  	NNSPAIKRVPASWMKISGTKKVSRFHTPEVVKMIRQRDQHREALAAACDKAFLALQAEIA
          	*.:. :::***** *******:****** *:::* :****:*:**:*** **  : :.**

MG09306.1 	DQYQPLRDAVSSLATLDCLLSFAQVAALPGYSKPIILPDSHPPTIAVAGGRHPIAEHTLP
NCU08115.1	GEYQPLRDAVSSLSTLDCLLSLSTVAALPGYTKPTFLPSSHPSFLSITEGRHPIAEHLLP
FG07112.1 	SDYQPLRDAVSALATLDCALSLSKVAAQPGYSRPSFLPSSTDPTISITNGRHAIAEHTLE
AN3749.1  	TNYQALRDCVQSLATLDCLVSLATLASQPGYVKP---EYTEETCIHVEQGRHPMVEQLLL
          	 :**.***.*.:*:**** :*:: :*: *** :*     :  . : :  ***.:.*: * 

MG09306.1 	SGYIPFSTTL-------SSPAP----------LAHLVTGPNMGGKSSFVRALALLVLLAQ
NCU08115.1	NGYIPFTMSLGTLSSSASSPDPNPTSPSGKPALAQLITGPNMGGKSSYTRAVALLVLLAQ
FG07112.1 	GGYIPFSTTL-------AHPSP----------LAHLITGPNMGGKSSFVRALALIVLLSQ
AN3749.1  	DSYVPNDINLD------SSKTR-----------ALLVTGPNMGGKSSYVRQVALIAIMGQ
          	..*:*   .*.      :               * *:**********:.* :**:.::.*

MG09306.1 	VGSFVPADSLRLTLSDAIYTRMGASDNLFAGESTFMVEVGETAAILRTATPRSLVLLDEL
NCU08115.1	IGSFVPATSMSLTLSDAIFTRMGARDNLFKGESTFMVEVSETAAILRQATPRSLVVLDEL
FG07112.1 	VGSYVPADSLSLTLCDAIHTRTGARDNLFAGESTFMVEVSETARILRSAGPRSLVILDEL
AN3749.1  	IGSYVPAQAAKLGMLDAVFTRMGAFDNMLAGESTFMVELSETADILKQATPRSLVILDEL
          	:**:*** :  * : **:.** ** **:: ********:.*** **: * *****:****

MG09306.1 	GRGTSTHDGAAIAHAVLDHVVRNTRCLTLFITHYQSLARVAEGLGTGL-------VRNVH
NCU08115.1	GRGTSTHDGRAIAGAVLEYVVRDVGCLMLFVTHYQDLAGVAEGLTVGEGEEKRRGVECVH
FG07112.1 	GRGTSTHDGAAIAQAVLQHVVTETRCLTLFITHYQNLARVAEGLDG---------VKNVH
AN3749.1  	GRGTSTHDGVAIAQAVLDYMVRSIRSLTLFITHYQHLSAMVHSFPDGE-------LRNVH
          	********* *** ***:::* .  .* **:**** *: :...:  .        :. **

MG09306.1 	MRFTSSRD----------DNDDGDKDQ----DDDVGENITFLYEVADGVAHRSYGLNVAR
NCU08115.1	MRFASNKSRTSMDDDAMEVDGDGDGQEGAGADKDEEEEITFLYDLAPGVAHRSYGLNVAR
FG07112.1 	MKFKAEK------------GEDGE------------EEVTFLYEVGEGVAHRSYGLNVAR
AN3749.1  	MRFSESGTG-------------------------ADEDITFLYEIGEGVAHRSYGLNVAR
          	*:*  .                              *::****::. *************

MG09306.1 	LARIPRKILEVAARKSRKMEEDVRTRRLRSATKLLEAVCRG----DGSDDQLEHLLSSIE
NCU08115.1	LARIPRKVLEVAARKSSELEKEVRAKRIKGAMGLVGGVLYGGGAPGDQEEKLEQLVGLVE
FG07112.1 	LARIPKKVIDVAALKSSQIEQEMKMRRLRGVCRALSEVM------DDGTDHLDQIVSSIE
AN3749.1  	LANLPAPLLEMAKQKSAELEEKIRRRRLAGFVAAVGAVVQSN---QADESVIERLVSSME
          	**.:*  ::::*  ** ::*:.:: :*: .    :  *  ..       . :::::. :*

MG09306.1 	QL
NCU08115.1	QL
FG07112.1 	QL
AN3749.1  	EL
          	:*
```
